# Effects of microencapsulated essential oils and seaweed meal on growth performance, digestive enzymes, intestinal morphology, liver functions, and plasma biomarkers in broiler chickens

**DOI:** 10.1093/jas/skaf092

**Published:** 2025-03-23

**Authors:** Ahmed A Elolimy, Mosaad M Hashim, Salah A Elsafty, Abdel Rahman Y Abdelhady, Stéphanie Ladirat, Mohamed Shourrap, Mahmoud Madkour

**Affiliations:** Department of Integrative Agriculture, College of Agriculture and Veterinary Medicine, United Arab Emirates University, Al Ain P.O. Box 15551, Abu Dhabi, United Arab Emirates; Applied Feed Research House, AFRH, Orabi Community, Al Obour City, Qalyobia, Egypt; Applied Feed Research House, AFRH, Orabi Community, Al Obour City, Qalyobia, Egypt; Department of Poultry Production, Faculty of Agriculture, Ain Shams University, Hadayek Shoubra, Cairo 11241, Egypt; Department of Poultry Production, Faculty of Agriculture, Ain Shams University, Hadayek Shoubra, Cairo 11241, Egypt; NUQO S.A.S, Annecy, France; Department of Poultry Production, Faculty of Agriculture, Ain Shams University, Hadayek Shoubra, Cairo 11241, Egypt; Animal Production Department, National Research Centre, Giza 12622, Egypt

**Keywords:** broiler, gene, gut, liver, phycogenic, phytogenic

## Abstract

Globally, poultry production has increased to meet the demand for animal protein. Traditionally, antibiotic growth promoters have been used to enhance growth performance and prevent infections in commercial poultry practices. However, concerns regarding antimicrobial resistance have triggered interest in alternative solutions, such as essential oils (**EOs**) and seaweed additives. The aim of the current study was to assess the impact of a microencapsulated blend of EOs (cinnamaldehyde, eugenol, and thymol) and *Ascophyllum nodosum* seaweed meal on growth performance, intestinal function, blood biomarkers, and hepatic gene expression in broiler chickens. A total of 440 Arbor Acres chicks were randomly assigned to either a control (CON) or treatment (NEX) group. Each treatment was divided into 11 replicates (20 birds per replicate). NEX chicks were supplemented with 100 mg/kg feed containing a microencapsulated blend of EOs (cinnamaldehyde, eugenol, and thymol) and *Ascophyllum nodosum* seaweed meal. Data were analyzed using the UNIVARIATE procedure in SAS software. Each replicate was considered an experimental unit. Over a 35-d period, NEX supplementation improved the feed conversion ratio (*P* = 0.02), reduced mortality rate (*P* = 0.01), and increased the European performance efficiency factor. No differences in carcass traits were observed between the 2 treatments (*P* > 0.05). Jejunal digestive enzyme activities, particularly those of amylase and lipase, were higher in NEX birds (*P* < 0.05) and correlated with morphometric parameters, such as villus height (*P* = 0.04) and muscular layer thickness (*P* < 0.01). Gene expression analysis revealed the upregulation of key genes related to nutrient transporters (solute carrier family 5 member 1 gene (*SLC5A1*), solute carrier family 1 member 1 gene (*SLC1A1*), solute carrier family 15 member 1 gene (*SLC15A1*)) in the jejunum (*P* < 0.05) and lipid metabolism (peroxisome proliferator-activated receptor alpha gene (*PPARA*) and microsomal triglyceride transfer protein gene (*MTTP*)) in the liver (*P* < 0.05) of NEX-supplemented birds. NEX treatment altered plasma biomarkers, including increased glucose (*P* < 0.01), insulin (*P* < 0.01), and protein profiles (*P* < 0.05) but decreased low-density lipoprotein cholesterol (*P* = 0.03), suggesting enhanced metabolic health. NEX supplementation improved growth performance, economic efficiency, intestinal morphology, digestive enzyme activity, liver function, and metabolic biomarkers in broiler chickens.

## Teaser

This study highlights how a blend of microencapsulated essential oils and seaweed meal can effectively enhance growth performance, gut health, and metabolic functions in broiler chickens, offering a natural alternative to antibiotic growth promoters.

## Introduction

Poultry is the most consumed source of animal protein worldwide (Whitton et al., 2021). Global poultry production has increased 5-fold over the past 50 yr to meet the demand for meat (FAO, 2020). To enhance growth performance and prevent infections, commercial poultry farms commonly add subinhibitory doses of antibiotics such as bacitracin to poultry feeds as antimicrobial growth promoters (**AGPs**) (Abdulrahim et al., 1999; Knarreborg et al., 2002). However, the overuse of these subtherapeutic antibiotic levels can lead to negative outcomes, such as an increase in antimicrobial resistance (de Mesquita Souza Saraiva et al., 2022). This has prompted the poultry industry to adopt antibiotic-free rearing practices (Lyte et al., 2024). Nonetheless, the removal of AGPs from feed can reduce growth performance and increase the incidence of bacterial infections in fast-growing, modern broiler chickens (Yang et al., 2021). Consequently, the poultry industry is exploring alternatives to AGPs (Madkour et al., 2024b).

Essential oils (**EOs**) are phytogenic, volatile oils derived from aromatic plants and are promising alternatives to AGPs (Zhu et al., 2021). Cinnamaldehyde, eugenol, and thymol are EOs that are mainly extracted from plants and possess antimicrobial, anti-inflammatory, and antioxidant properties. Cinnamaldehyde is found in the bark of the cinnamon tree (*Cinnamomum zeylandicum*) (Aljazzar et al., 2022), eugenol is a phenolic compound found in cloves (*Syzgium aromaticum*) (Aljazzar et al., 2022), and thymol is found in thyme (*Thymus vulgaris L.*) (Hassanin et al., 2024). Recent studies have indicated that EOs enhance growth performance and overall health of broiler chickens (Ibrahim et al., 2021; Zaazaa et al., 2022; Madkour et al., 2024a). These benefits are attributed to the increased secretion of digestive enzymes, improved intestinal integrity, and enhanced nutrient absorption, eventually providing additional nutrients and energy to birds (Jin et al., 2020; Abd El-Hack et al., 2022). However, the use of EOs as feed additives is limited due to their instability during long-term storage, feed processing, and passage through the digestive system (Yuliani et al., 2018; Kang et al., 2024).

Initially, EOs were added to feed without protection (Hernández et al., 2004; Mitsch et al., 2004). Given their high volatility and sensitivity to environmental conditions, this often led to the degradation or early absorption of EOs before they reached the absorption sites of the small intestine (Stamilla et al., 2020; Oladokun et al., 2021). To address this issue, microencapsulation technology has been developed to protect EOs with a fat-based matrix encapsulation to maintain their integrity until delivery (Bosetti et al., 2020; Stamilla et al., 2020; Mullenix et al., 2024). Microencapsulation is particularly beneficial for EOs and seaweed because microencapsulation prevents their degradation during feed processing and storage, ensures targeted release in the small intestine, and enhances their bioavailability, thereby maximizing their beneficial effects on digestion, nutrient absorption, and gut health (Tolve et al., 2021). This approach has increased the stability of EOs and allows targeted release in the small intestine rather than in the stomach (Abdelli et al., 2020; Mullenix et al., 2024).

*Ascophyllum nodosum*, a phycogenic brown seaweed, is rich in antioxidants and polysaccharides that are beneficial for broiler chicken growth and health because of its antioxidative, anti-inflammatory, antibacterial, and immunomodulatory properties (Allen et al., 2001; Coudert et al., 2020; El-Hady et al., 2022). Studies have shown that *Ascophyllum nodosum* meal or extract improves growth performance and mitigates heat stress by altering plasma enzyme activity in broiler chickens (Akinyemi and Adewole, 2022; Archer, 2023). Other studies have shown that *Ascophyllum nodosum* extract reduces the colonization of pathogenic bacteria such as *Campylobacter jejuni* in the cecum of young broiler chicks (Sweeney et al., 2016; Bonifait et al., 2022). In a recent study, Mullenix et al. (2024) assessed the effects of dietary supplementation with a microencapsulated blend of phytogenic EOs (cinnamaldehyde, eugenol, and thymol) and phycogenic *Ascophyllum nodosum* meal in male broiler chicks at 2 different doses: 100 mg/kg of feed from days 1 to 28, followed by 75 g/ton from days 28 to 40, or 100 mg/kg of feed from days 1 to 40. The authors demonstrated that supplementation with 100 mg/kg of feed from days 1 to 40 resulted in increased body weight (**BW**) gain, enhanced feed conversion ratio (**FCR**), and reduced bone lesions.

However, there are no available studies on the impact of microencapsulated phytogenic–phycogenic blends on intestinal function. Therefore, the current study aimed to explore the impact of dietary supplementation with a microencapsulated blend of EOs (cinnamaldehyde, eugenol, and thymol) and *Ascophyllum nodosum* seaweed meal on growth performance, intestinal function, circulatory biomarkers, and liver gene expression in broiler chickens.

## Materials and Methods

The experiment was conducted at the Applied Feed Research House (**AFRH**), Orabi Community, Qalyobia, Egypt. All procedures were approved by the Medical Research Ethics Committee of the National Research Centre, Giza, Egypt (approval number 14810112022).

### Experimental design and treatments

A total of 440 one-day-old unsexed Arbor Acres broiler chicks were obtained from a commercial hatchery and housed in an environmentally controlled facility at the AFRH. Birds were randomly assigned to 1 of 2 dietary treatments: 1) a control group (CON) receiving a standard corn–soybean meal-based diet, and 2) a supplemented group (NEX) receiving the same diet supplemented with 100 mg/kg of a microencapsulated blend of EOs (10% cinnamaldehyde, 1% eugenol, 10% thymol) and 10% *Ascophyllum nodosum* seaweed meal according to the manufacturer (NUQO NEX, NUQO SAS, Annecy, France) and Mullenix et al. (2024) recommendations. Each treatment was replicated 11 times with 20 birds per replicate. The sample size (440 birds, 11 replicates per group, and 20 birds per replicate) was determined based on power calculations from previous studies evaluating feed additives in broilers, ensuring statistical robustness in detecting treatment effects on performance and physiological parameters (Huang et al., 2024; Toschi et al., 2024). Diets were formulated to meet or exceed nutrient recommendations of commercial breeder standards (ArborAcres, 2022) (Table 1).

**Table 1.** Ingredients and nutrient levels of the experimental basal diet

Composition	Starter 0 to 14 d	Grower 15 to 28 d	Finisher 28 to 35 d
Ingredients			
Yellow Corn	54.490	60.708	65.893
Soybean meal 46%	36.091	28.290	21.800
Corn gluten meal 60%	1.720	2.490	3.514
Soybean oil	0.500	1.000	1.500
Wheat middlings	4.000	4.000	4.000
Calcium carbonate	0.900	1.100	1.065
Mono-calcium Phosphate	1.076	1.050	0.956
Na bicarbonate	0.093	0.140	0.142
Salt	0.220	0.190	0.196
Lysine	0.213	0.326	0.319
dl-Methionine	0.262	0.262	0.174
l-Threonine	0.035	0.044	0.041
β-Mannanase enzyme^1^	0.030	0.030	0.030
Choline	0.050	0.050	0.050
Phytase enzyme^2^	0.010	0.010	0.010
NSP enzymes^3^	0.010	0.010	0.010
Premix^4^	0.300	0.300	0.300
Total	100.000	100.000	100.000
Chemical composition (%)			
AME (kcal)	2,950	3,050	3,150
CP	22.50	20.00	18.00
Ca (total)	0.90	0.90	0.85
Phosphorus (total)	0.50	0.48	0.45
Lysine (total)	1.36	1.25	1.09
Methionine (total)	0.60	0.58	0.47

^1^Hemicell, Elanco, Indiana, USA.

^2^Quantum Blue, AB Vista, Wiltshire, UK.

^3^Xylamax, BRI, North Carolina, USA.

^4^Each 3 kg of premix contain: vitamin A: 12,000,000 IU; vitamin D3: 5,000,000 IU; vitamin E: 10,000 mg; vitamin K3: 2,000 mg; vitamin B1: 1,000 mg; vitamin B2: 5,000 mg; vitamin B6: 1,500 mg; vitamin B12: 10 mg; Biotin: 50 mg; pantothenic acid: 10,000 mg; nicotinic acid: 30,000 mg; folic acid: 1,000 mg; and trace minerals: Mn: 60,000 mg; Zn: 50,000 mg; Fe: 30,000 mg; Cu: 10,000 mg; I: 1,000 mg; Se: 100 mg, and Co: 100 mg.

### Birds management

Chicks were housed on floor pens (1.0 m × 1.0 m per replicate) with ad libitum access to water and feed. The temperature was maintained at 32 °C for the first 3 d, then gradually reduced by 0.5 °C per day until reaching 22 to 24 °C. Lighting was provided for 24 h on the first day, followed by a 23-h light, 1-h dark cycle. Health monitoring was performed twice daily, and any mortalities were recorded.

### Growth performance

The initial body weight (**IBW**) of each chick was recorded at the beginning of the experiment (and each week). Subsequently, live body weight (**BW**) was measured weekly using an electronic weighing balance to calculate the average daily gain (**ADG**) per bird:

ADG (g/d) = [BW (g)—IBW (g)]/7

Weekly feed intake (**FI**) per pen was calculated as the difference between the weight of feed offered at the beginning of the week and the weight of feed left over at the end of the week. The difference was divided by the total number of birds in the pen and further divided by 7 to determine the average daily FI per bird. ADG and FI were used to calculate the FCR of each bird every 7 d for the experimental period:

FCR = FI (g) / ADG (g)

Mortality was recorded daily for each pen and used to adjust the ADG, FI, and FCR per bird. European production efficiency factor (EPEF) and mortality percentage were calculated as previously described by Biesek et al. (2022).

### Sample collections

On day 35, 20 birds (1 bird per pen from 10 pens per treatment) were randomly selected and processed by trained personnals. Birds were chosen based on average BW to ensure they were representative of each replicate’s performance. Birds showing signs of illness or extreme outliers in weight were excluded to avoid bias. Birds were weighed individually and humanely slaughtered by severing the jugular vein. Blood samples were collected in a 10-mL tube containing sodium heparin and then centrifuged at 3,000 × *g* at 4 °C for 20 min to obtain the plasma (El-Wardany et al., 2016; Alagawany et al., 2022; Reda et al., 2022). The plasma samples were stored at − 80 °C before further analysis. The concentrations of glucose, insulin, total protein, albumin, globulin, alpha 1 globulin, alpha 2 globulin, beta 1 globulin, beta 2 globulin, gamma globulin, creatinine, uric acid, alanine transaminase (**ALT**), aspartate aminotransferase (**AST**), alkaline phosphatase (**ALP**), total cholesterol, triglycerides, high-density lipoprotein (**HDL**) cholesterol, low-density lipoprotein (**LDL**) cholesterol, and very low-density lipoprotein (**VLDL**) cholesterol were analyzed according to the manufacturer’s instructions (Spinreact Co., Girona, Spain).

All the birds were subject to inspection immediately after slaughter. Approximately 1 g of liver tissue was collected, gently flushed with 4 °C phosphate-buffered saline, snap-frozen in liquid nitrogen, and stored at −80 °C for gene expression analysis. The jejunum plays a central role in nutrient absorption and intestinal health in broilers and acts as a crucial site for the digestion and absorption of nutrients, such as carbohydrates (Wang et al., 2022), proteins (Barekatain et al., 2021), lipids (Cordero et al., 2023), vitamins (Calik et al., 2022), and minerals (Wang et al., 2023). Therefore, a 1-cm segment from the proximal jejunum was harvested, gently flushed with ice-cold PBS to remove contents, then rapidly stored in liquid nitrogen, and transferred to a −80 °C freezer for gene expression analysis. Additionally, a 3-cm section of the proximal jejunum was collected for tissue morphology assessment. Jejunal contents were also collected, snap-frozen in liquid nitrogen, and stored at −80 °C for digestive enzyme analyses.

### Total RNA isolation and quantitative real-time polymerase chain reaction (qRT-PCR)

Ten milligrams of jejunum or frozen liver tissue were immediately placed in TRIZOL reagent (Invitrogen Life Technologies, Palo Alto, CA, USA) and homogenized to extract total RNA, following the manufacturer’s recommendations (Hemida et al., 2023). The RNeasy Mini Kit (Qiagen, Valencia, CA, USA) was used to purify total RNA. RNA quantity and quality were assessed using NanoDrop 1000 (Nanodrop Technologies, Rockland, DE, USA) and agarose gel electrophoresis (Thermo Scientific, Wilmington, DE, USA), respectively. The total purified RNA was converted into cDNA using the RevertAid First Strand cDNA Synthesis Kit (Thermo Fisher Scientific, Cat. #K1621) according to the manufacturer’s instructions. The cDNA was diluted 10-fold with DNase/RNase-free water.

The conventional PCR approach was used to optimize the annealing temperatures for each set of primers (Table 2) and rule out the existence of nonspecific products. These products were then examined using 2% agarose gel electrophoresis. A Quant Studio 5 Real-Time PCR System (Applied Biosystems, Foster City, CA, USA) was used for qPCR analysis. The thermal cycling profiles started with a denaturation step lasting 10 min at 95 °C, then went through 40 cycles with the following parameters: denaturation for 30 s at 95 °C, annealing for 15 s at the designated annealing temperature for each primer pair, and extension for 30 s at 72 °C. The total reaction volume for qPCR was 15 µL, containing 7.5 µL of a 2X SYBR Green PCR Master Mix kit (Thermo Fisher Scientific, Cat. #K0251), DNase/RNase-free water, and 0.3M forward and reverse specific primers for each gene (Table 2), and 2 µL of cDNA template. Chicken *β-actin* was used as an internal control.

**Table 2.** Primer sequences for fluorescence-based quantitative real-time PCR

Genes	GenBank number		Primer sequences (5’→ 3’)	Reference
*SLC5A1*	NM_001293240	F R	CAGAACGTTTGAGGGCTTTGT AGCAAGTGGAGCCAATCAGA	Su et al. (2021)
*SLC2A2*	NM_207178.1	F R	CAGGAACGTTGGTCCTCTCC GCGCCCATAGTGTGCTTCTA	Du et al. (2023)
*SLC1A1*	XM_424930	F R	TGCTGCTTTGGATTCCAGTGT AGCAATGACTGTAGTGCAGAAGTAATATATG	Gilbert, et al. (2007)
*SLC7A1*	NM_001145490.2	F R	CTCTGGCTTGGTGGTGAACATCTC GCGTGCTTGGCTTGAGGGTAG	Wu et al. (2024)
*SLC15A1*	NM_204365.1	F R	TCCCATGGAGTCAACAGGCT GCTAGAAACAATGCCGGCTG	Wang et al. (2021)
*PPARA*	AF163809	F R	CAAACCAACCATCCTGACGAT GGAGGTCAGCCATTTTTTGGA	Nguyen et al. (2015)
*PPARG*	NM_001001460	F R	CACTGCAGGAACAGAACAAAGAA TCCACAGAGCGAAACTGACATC	Nguyen et al. (2015)
*FASN*	JO3860	F R	ACTGTGGGCTCCAAATCTTCA CAAGGAGCCATCGTGTAAAGC	Nguyen et al. (2015)
*MTTP*	NM001109784	F R	GACGGTACACTGCGAGGAGA GCCTGAGGAATCAGATGCAG	Lu et al. (2020)
*SCD*	NM_204890	F R	TCCCTTCTGCAAAGATCCAG TCCCGTGGGTTGATGTTCTG	Suzuki et al. (2019)
*β-actin*	NM_205518.1	F R	AATGGCTCCGGTATGTGCAA GGCCCATACCAACCATCACA	Lu et al. (2020)

Abbreviations: *MUC2*, mucin2; *SLC5A1,* solute carrier family 5 member 1; *SLC2A2,* solute carrier family 5 member 1; *SLC1A1*, solute carrier family 1 member 1; *SLC7A1*, solute carrier family 7 member 1; *SLC15A1*, solute carrier family 15 member 1; *PPARA*, peroxisome proliferator-activated receptor alpha; *PPARG*, peroxisome proliferator-activated receptor gamma; *FASN*, fatty acid synthase; *MTTP*, microsomal triglyceride transfer protein; *SCD*, stearoyl-CoA desaturase.

Dissociation curves were constructed to validate the quality of the data at the end of amplification. All qRT-PCR experiments were performed in triplicate. The values of the average cycle threshold (Ct) were determined and Delta-Ct scores for gene transcripts in each sample were normalized using Delta-Ct scores for *β-actin* and expressed as the fold change in gene expression using the equation, 2^−ΔΔCT^ (Rao et al., 2013).

### Jejunum morphometric measurements

Three cm of the proximal jejunum were rinsed with saline (0.85 % NaCl) and fixed in a 12% neutral formalin solution. Thin transverse sections (4 to 5 µm) were cut using a microtome, and mounted on glass slides (9 sections/sample/slide), then stained with the ordinary hematoxylin and eosin stain (H & E) procedures according to the method of Bancroft and Gamble (2021). Histological sections were examined using a routine light microscope (OPTIKA, Model B-193) equipped with a digital microscope camera (OPTIKA, Model C-B). Images were captured under a low magnification of ×4 for histomorphometric analysis (Abdel-Fattah et al., 2024). The villus height (measured from the tip to the villus–crypt junction) from 3 to 5 intact villi per section, villus width at one-third and 2-thirds of the villus length, and crypt depth (from the base of the villus to the submucosa) were assessed using the image analysis software, OTIKA PROVIEW (version x86). The villus height-to-crypt depth ratio was calculated for each segment. Additionally, the muscular layer thickness was measured as the vertical distance from the epimysium to the submucosal layer.

### Digestive enzyme activity

Jejunal contents were used to determine the activity of digestive enzymes. Amylase and lipase activities were quantified using commercial colorimeter kits (Spinreact, Santa Coloma, Spain), according to the manufacturer’s instructions. Trypsin activity was quantified using an enzyme-linked immunosorbent assay kit (Assay Genie, Dublin, Ireland), according to the manufacturer’s instructions.

### Statistical analyses

All data were analyzed using the UNIVARIATE procedure in SAS 9.4 (SAS Institute Inc., Cary, NC, USA), accompanied by a post hoc Tukey’s test to determine the differences between the 2 treatments. Replicates were defined as experimental units for the trial. The results are presented as means with standard errors of the mean. Statistical significance was considered at *P* ≤ 0.05, whereas a tendency was determined at *P* ≤ 0.10.

## Results

**Figure 1.**
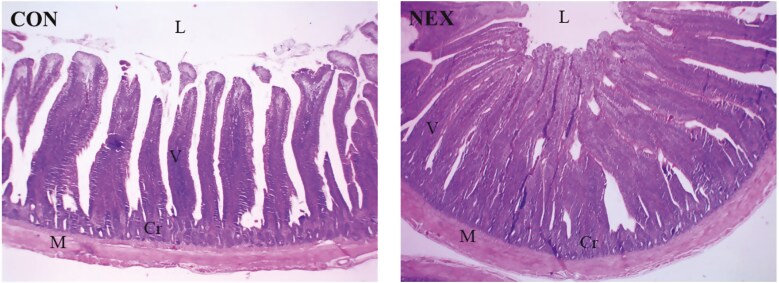
Representative photomicrograph of the (40×) magnification H&E stained jejunum segment in 35-d-old Arbor Acres broilers fed either a control basal diet (CON) or CON supplemented with NUQO NEX (NEX). L: lumen; V: villus; M: mucosa; Cr: crypts.

The NEX treatment improved FCR (*P* = 0.04) at 8 to 14 d of age compared to the CON birds (Table 3). Furthermore, NEX supplementation tended to decrease FI (*P* = 0.06) and enhance FCR (*P* < 0.01) at 29 to 35 d of age (Table 3). The experimental period from 1 to 35 d of age indicated that NEX treatment improved the FCR (*P* = 0.02), reduced mortality (*P* = 0.01), and increased the EPEF (*P* = 0.04) (Table 3). Digestive enzyme activity in the jejunum indicated that NEX birds had improved amylase activity (*P* = 0.03) and tended to have increased lipase activity (*P* = 0.08) compared to the CON (Table 4). The morphometric results indicated that NEX treatment increased muscle layer thickness (*P* < 0.01), crypt depth (*P* = 0.04), and villus height (*P* = 0.04) (Fig. 1 and Table 4). In contrast, NEX treatment tended to decrease villus height and crypt depth (*P* = 0.07) compared to CON birds (Fig. 1 and Table 4).

**Table 3.** Growth performance and mortality rate of 35-d-old Arbor Acres broilers fed either a control basal diet (CON) or CON supplemented with NEX

Items	CON	NEX	SEM^1^	*P*-value
1 to 7 d				
BW at d 7, g	189.70	190.00	2.65	0.94
ADG, g	21.31	21.39	0.40	0.88
FI, g	24.76	24.64	0.44	0.83
FCR	1.16	1.15	0.05	0.55
8 to 14 d				
BW at d 14, g	510.10	508.60	8.06	0.88
ADG, g	45.77	45.52	0.87	0.83
FI, g	54.44	54.09	0.81	0.75
FCR	1.22^a^	1.19^b^	0.01	0.04
15 to 21 d				
BW at d 21, g	999.70	981.20	11.00	0.30
ADG, g	69.95	67.52	1.34	0.21
FI, g	95.84	93.04	2.37	0.33
FCR	1.37	1.38	0.01	0.52
22 to 28 d				
BW at d 28, g	1558.30	1548.00	18.5	0.67
ADG, g	79.79	80.97	2.07	0.65
FI, g	126.40	129.60	3.20	0.41
FCR	1.59	1.60	0.02	0.56
29 to 35 d				
BW at d 35, g	2199.80	2202.00	30.30	0.96
ADG, g	91.64	93.42	2.32	0.58
FI, g	172.50	162.80	3.79	0.06
FCR	1.89^a^	1.74^b^	0.03	<0.01
1 to 35 d				
ADG, g	61.69	61.76	0.87	0.95
FI, g	95.10	92.83	1.28	0.18
FCR	1.513^a^	1.476^b^	0.01	0.02
Mortality %	2.73^a^	1.36^b^	0.20	0.01
EPEF %^2^	404^b^	421^a^	6.00	0.04

^1^Standard error of means.

^2^European performance efficiency factor.

Different letters indicate significant differences at *P* < 0.05. Tukey–Kramer HSD test was used when appropriate.

**Table 4.** Effects of NUQO NEX on the jejunum digestive enzymes activity and histological parameters in broiler chickens at 35 d of age

Item	CON	NEX	SEM^1^	*P*-value
Digestive enzymes activity				
Amylase, U/L	382.20^b^	514.90^a^	40.92	0.03
Lipase, U/L	125.30	162.50	14.75	0.07
Protease, ng/mL	9.55	9.64	0.93	0.95
Histological parameters				
Muscular layer thickness, µm	165.60^b^	195.70^a^	3.97	<0.01
Crypt depth, µm	159.40^b^	169.20^a^	3.46	0.04
Villus height, µm	1,004.70^b^	1,046.70^a^	14.55	0.04
Villus width, µm	201.30	196.00	5.25	0.47
Villus height:crypt depth	6.49	6.16	0.15	0.07

^1^Standard error of means.

Different letters indicate significant differences at *P* < 0.05. Tukey–Kramer HSD test was used when appropriate.

Table 5 presents the effects of NEX supplementation on intestinal and hepatic gene expression. The expression levels of solute carrier family 5 member 1 gene (*SLC5A1*) (*P* = 0.04), solute carrier family 1 member 1 gene (*SLC1A1*) (*P* < 0.01), and solute carrier family 15 member 1 gene (*SLC15A1*) (*P* = 0.02) in the jejunum were higher in NEX group (Table 5). In the liver, NEX treatment upregulated peroxisome proliferator-activated receptor alpha gene (*PPARA*) (*P* < 0.01) and microsomal triglyceride transfer protein gene (*MTTP*) (*P* = 0.04) compared to the CON birds (Table 5). The plasma results indicated that dietary NEX supplementation increased glucose (*P* < 0.01), insulin (*P* < 0.01), total protein (*P* = 0.02), globulin (*P* = 0.03), alpha 1 globulin (*P* = 0.01), alpha 2 globulin (*P* = 0.03), beta 1 globulin (*P* = 0.02), beta 2 globulin (*P* = 0.04; Table 6). NEX supplementation decreased albumin:globulin (*P* = 0.04), LDL cholesterol (*P* = 0.03), and tended to decrease ALT levels (*P* = 0.08) (Table 6).

**Table 5.** Effects of NUQO NEX supplementation on the expression of key genes in small intestinal functions and liver energy metabolism in broiler chickens at 35 d of age

Tissue	Gene	CON	NEX	SEM	*P*-value
Jejunum	*SLC5A1*	0.97^b^	1.34^a^	0.14	0.04
	*SLC2A2*	0.87	1.17	0.31	0.40
	*SLC1A1*	1.43^b^	4.32^a^	0.74	<0.01
	*SLC7A1*	0.82	0.83	0.15	0.99
	*SLC15A1*	1.35^b^	3.14^a^	0.58	0.02
Liver	*PPARA*	1.09^b^	2.33^a^	0.16	<0.01
	*PPARG*	1.25	1.52	0.24	0.38
	*FASN*	1.13	1.07	0.17	0.81
	*MTTP*	1.04^b^	2.06^a^	0.30	0.04
	*SCD*	1.19	0.97	0.22	0.47

Abbreviations: *SLC5A1,* solute carrier family 5 member 1; *SLC2A2,* solute carrier family 5 member 1; *SLC1A1*, solute carrier family 1 member 1; *SLC7A1*, solute carrier family 7 member 1; *SLC15A1*, solute carrier family 15 member 1; *PPARA*, peroxisome proliferator-activated receptor alpha; *PPARG*, peroxisome proliferator-activated receptor gamma; *FASN*, fatty acid synthase; *MTTP*, microsomal triglyceride transfer protein; *SCD*, stearoyl-CoA desaturase.

**Table 6.** Effects of NUQO NEX supplementation on plasma indices in broiler chickens at 35 d of age

Item	CON	NEX	SEM^1^	*P-*value
Glucose, mg/dL	62.42^b^	113.00^a^	11.12	<0.01
Insulin, ng/mL	1.44^b^	3.57^a^	0.56	<0.01
Total protein, g/dL	3.33^b^	3.47^a^	0.04	0.02
Albumin, g/dL	1.70	1.65	0.03	0.20
Globulin, g/dL	1.66^b^	1.83^a^	0.05	0.03
Albumin:globulin	1.04^a^	0.91^b^	0.04	0.04
Alpha 1 globulin, g/dL	0.06^b^	0.10^a^	0.01	0.01
Alpha 2 globulin, g/dL	0.11^b^	0.16^a^	0.01	0.03
Beta 1 globulin, g/dL	0.11^b^	0.13^a^	0.01	0.02
Beta 2 globulin, g/dL	0.14^b^	0.19^a^	0.02	0.04
Gamma globulin, g/dL	0.25	0.23	0.02	0.37
Creatinine, mg/dL	0.42	0.45	0.02	0.40
Uric acid, mg/dL	6.14	6.00	0.47	0.80
ALT^2^, U/L	18.81	14.58	1.78	0.08
AST^3^, U/L	109.30	98.14	7.19	0.25
ALP^4^, U/L	855.40	824.30	43.97	0.60
Total cholesterol, mg/dL	212.00	198.10	9.76	0.28
Triglycerides, mg/dL	134.10	128.10	3.89	0.24
HDL^5^ cholesterol, mg/dL	50.72	52.96	3.40	0.61
LDL^6^ cholesterol, mg/dL	184.00^a^	162.00^b^	6.92	0.03
VLDL^7^ cholesterol, mg/dL	20.03	20.07	2.22	0.99

^1^Standard error of means.

^2^Alanine transaminase.

^3^Aspartate aminotransferase.

^4^Alkaline phosphatase.

^5^High-density lipoprotein.

^6^Low-density lipoprotein.

^7^Very low-density lipoprotein.

Different letters indicate significant differences at *P* < 0.05. Tukey–Kramer HSD test was used when appropriate.

## Discussion

The current study focused on the combined effects of microencapsulated EOs and seaweed meal. However, the individual contributions of each component on growth performance and gut health have been reported early. Previous studies have shown that EOs such as cinnamaldehyde, eugenol, and thymol possess antimicrobial, anti-inflammatory, and antioxidant properties, which can enhance gut health and nutrient absorption (Zhu et al., 2021; Abd El-Hack et al., 2022). Similarly, seaweed meal (*Ascophyllum nodosum*) has been reported to improve growth performance and mitigate stress in broilers due to its high antioxidant and polysaccharide content (Akinyemi and Adewole, 2022; Archer, 2023). The current study indicated that supplementing broiler chickens with 100 mg of NEX per kg of feed tended to reduce FI during days 29 to 35 of the experimental period and improved FCR over the first 35 d of age. Similarly, a recent study found that dietary supplementation with 100 mg/kg feed of the same microencapsulated EO–seaweed blend (containing cinnamaldehyde, eugenol, thymol, and *Ascophyllum nodosum* meal) reduced FI between days 15 to 28 and improved FCR throughout the 40-d rearing period in male broilers (Mullenix et al., 2024).

A meta-analysis conducted by Irawan et al. (2021) evaluated the effects of dietary EOs on broiler growth. The study analyzed 41 articles with 55 experiments and 163 treatments wherein various types of EOs, such as cinnamaldehyde, eugenol, and thymol, whether used individually or in blends, decreased FI and improved FCR in broiler chickens (Irawan et al., 2021). Kumar et al. (2022) found that microencapsulated eugenol and garlic tincture reduced the FI in broiler chickens challenged with necrotic enteritis during the first 35 d of age. Pirgozliev et al. (2019) reported that an EO blend containing cinnamaldehyde, carvacrol, and capsicum oleoresin improved the FCR in male broilers. Other studies have shown that individual supplementation with eugenol and thymol enhanced the FCR compared to unsupplemented birds over the entire rearing period (Abdelli et al., 2021; Youssefi et al., 2023).

Several studies have highlighted the positive effects of *Ascophyllum nodosum* seaweed in improving FCR in broiler chickens under heat stress conditions (Choi et al., 2014; Akinyemi and Adewole, 2022; Archer, 2023). Therefore, the observed lower FI and better FCR in broilers fed NEX suggest that the microencapsulated EO-seaweed blend enhanced nutrient utilization efficiency, likely due to the stimulation of digestive enzymes (Upadhaya et al., 2019; Mohebodini et al., 2021), allowing birds to extract more energy and nutrients despite the reduction in FI. In the present study, the observed improvements in FCR with NEX were noted at 8 to 14 d and 29 to 35 d of age, resulting in improved FCR over the first 35 d of age. A recent study reported that the NEX treatment improved BWG and reduced FI during the grower (15 to 28 d) period, whereas it increased BWG during the finisher period (29 to 40 d), compared to the CON treatment in broiler chickens (Mullenix et al., 2024). These findings emphasize the need for further investigation into the timing and mechanisms underlying the impact of NEX treatment on growth performance of broilers.

The present study revealed that NEX supplementation reduced the mortality rate by 50% compared to the control birds (1.36% vs. 2.73%). Previous studies have reported similar results. Yang et al. (2020) demonstrated that encapsulated cinnamaldehyde reduced the mortality rate of broilers compared to unsupplemented broilers. Hassanin et al. (2024) found that thyme oil provided 100% protection from mortality in vaccinated birds challenged with Newcastle disease virus. Thymol supplementation reduced mortality rates in broilers under heat stress or when challenged with *Salmonella* or *Clostridium* (Du and Guo, 2021; Hamed et al., 2022; Senas-Cuesta et al., 2023). Choi et al. (2014) also found that dietary seaweed reduced the mortality rate of broilers compared to that of control broilers. Collectively, the reduction in mortality rate with NEX treatment may indicate better health and reduced stress in broiler chickens.

EPEF is a widely used indicator of economic efficiency and profitability in broiler operations (Weaver et al., 2020; Urban et al., 2023). The EPEF is calculated based on growth performance parameters, including livability, BW, and FCR (Huff et al., 2013). Broiler production is considered profitable when the EPEF exceeds 260 (Mavromati et al., 2018) and some reports suggest that maintaining profitability requires an EPEF of at least 300 (Biesek et al., 2022). A higher EPEF index indicates more economically efficient production and higher profits (Huff et al., 2013). In the present study, the NEX treatment showed an EPEF of 421, compared to 404 in the CON treatment, emphasizing its economic benefits.

The improvements observed in the NEX treatment, such as lower FI, better FCR, and reduced mortality, not only enhance performance, but also have the potential to reduce production costs by improving feed efficiency and minimizing losses. Therefore, the introduction of NEX into broiler production could be economically viable and could potentially increase profitability without substantially increasing production costs, making it a promising option for practical industrial application. Although the cost of NEX supplementation was not explicitly evaluated in this study, the improved feed efficiency and reduced mortality suggest potential economic benefits. Future studies should include a cost-benefit analysis to evaluate the economic viability of NEX supplementation in commercial broiler production.

The small intestine is the primary site of digestion and nutrient absorption (Lu et al., 2023). The digestive enzymes are essential for nutrient release during absorption. Therefore, higher digestive enzyme activity stimulates digestion and absorption capacity (Safari et al., 2024). Amylase and lipase are the main digestive enzymes involved in starch and lipid digestion in the intestine, respectively (Wan et al., 2017; Lin et al., 2023). The current study demonstrated that dietary supplementation with NEX promoted amylase and lipase activity in the jejunum. Previous studies have highlighted that EO blends containing thymol and *Ascophyllum nodosum* seaweed stimulates amylase activity in the intestinal digesta of broiler chickens (Lee et al., 2003; Akinyemi and Adewole, 2022). Other studies have found that providing broilers with plant extracts containing cinnamaldehyde and thymol induces lipase activity in the intestines of broilers (Jamroz et al., 2005; Hashemipour et al., 2013). It has been suggested that greater amylase and lipase activities in NEX birds improved starch and lipid digestion, ultimately extracting more energy for absorption and facilitating better feed digestibility and utilization, despite the lower FI (Akinyemi and Adewole, 2022).

Changes in intestinal digestive enzyme activity can lead to alterations in intestinal morphology, such as villus height and muscular layer thickness, which are crucial parameters for absorption capacity in the small intestine (Iji et al., 2001; Abd El-Hack et al., 2022). Histological analysis of the small intestine indicated that NEX supplementation increased villus height, muscular layer thickness, and crypt depth. In support of these findings, other studies have reported that dietary EOs and seaweed improve the histomorphological parameters in different bioregions of the small intestine, e.g., thymol, eugenol, and *Ascophyllum nodosum seaweed* increased villus height, muscular layer thickness, and crypt depth in the small intestine in broiler chickens (Du et al., 2016; Reis et al., 2018; Yang et al., 2018; Oretomiloye and Adewole, 2024). These morphometric alterations are indicators of improved health and function of the small intestine of broiler chickens (Sobolewska et al., 2017).

An increase in villus height reflects a higher recovery rate and greater surface area of the intestinal epithelial cells, leading to better nutrient uptake capacity (Samanya and Yamauchi, 2002; Cañedo-Castro et al., 2019). Thick muscular layers in the intestine, such as the *muscularis mucosae* and *muscularis externa*, induce villus contraction along the longitudinal and transverse folds of the mucosa (Sobolewska et al., 2017). This promotes proper intestinal motility, effective mixing of intestinal contents, and improved contact with digestive enzymes, leading to faster nutrient absorption (Sobolewska et al., 2017). Deeper crypts reflect more intense villus renewal in response to normal sloughing (Paiva et al., 2014). A study conducted by Xu et al. (2003) suggested that deeper crypts were associated with poor nutrient absorption and lower growth performance. In contrast, other studies have shown that deeper crypts can lead to increased intestinal secretion and absorption (Fan et al., 1997; Xu et al., 2003; Sobolewska et al., 2017), which is consistent with the findings of the present study wherein NEX treatment promoted higher digestive enzyme activity and nutrient absorption.

The current investigation revealed a tendency for villus height and crypt depth reduction, which might indicate higher maintenance needs and worse feed efficiency (Stokvis et al., 2022). The greater nutrient absorption and growth performance in the present study contradicts this theory, most likely as a result of the higher secretion of digestive enzymes and improved nutrient absorption. Collectively, NEX promoted intestinal secretion and absorption and improved feed efficiency and mortality.

The jejunum plays a key role in nutrient absorption in birds by expressing various enterocyte transporters (Biswas et al., 2022; Ghareeb et al., 2022). For example, glucose is transported into intestinal epithelial cells by the *SLC5A1* transporter at the apical brush border membrane, using an electrochemical sodium gradient created by Na^+^/K^+^-ATPase to move glucose into the cells against chemical gradients (Bell et al., 1990; Ariyo et al., 2023). Glucose transporters are essential for glucose uptake by enterocytes (Murugesan et al., 2014). Therefore, the abundance of the *SLC5A1* transporter is a crucial determinant of carbohydrate absorption efficiency. In the present study, the upregulation of *SLC5A1* in NEX broilers was likely due to an increase in intestinal amylase activity, which enhanced starch digestion and liberated more glucose for absorption. Thus, the upregulation of *SLC5A1* increases glucose uptake in the intestine (Agyekum et al., 2015), allowing NEX broilers to absorb extra glucose and support better feed efficiency, despite the lower FI in the NEX treatment compared to the control.

The jejunum is an active bioregion for the absorption of protein digestion products and amino acids (Tauqir, 2017). The *SLC1A1* gene encodes anionic amino acid (aspartate and glutamate) transporter (Gilbert et al., 2007) and initiates cell signaling via the mTOR pathway, which is a major metabolic pathway in protein synthesis (Fathima et al., 2022). Furthermore, *SLC1A1* provides energy to intestinal cells to support intestinal function and health (Iwanaga et al., 2005). *SLC15A1* encodes a transporter of small peptides and amino acids derived from the digestion of dietary proteins (Venardou et al., 2021). Thus, the upregulation of *SLC1A1* and *SLC15A1* genes with NEX supplementation in the current study elicited an increase in the influx and absorption of peptides into the intestinal epithelial cells, resulting in better protein utilization with NEX treatment.

Dietary glucose is the primary energy source for poultry (Reece, 2015). Irawan et al. (Irawan et al., 2021) conducted a meta-analysis of the effects of EOs in broilers using 41 articles. The authors reported that EOs, such as cinnamaldehyde, eugenol, and thymol, individually elevate blood glucose levels in broiler chickens (Irawan et al., 2021). In the present study, the increase in plasma glucose levels following NEX supplementation may be attributed to the synergistic effects of the combination of EOs and seaweed. This elevation in plasma glucose may be linked to enhanced carbohydrate digestion and absorption due to an increase in amylase activity, induction of the glucose transporter *SLC5A1*, and an increase in villus height in the small intestine. These alterations in the small intestine could contribute to the greater absorption of glucose reaching the circulation to provide a readily available energy source and support anabolic pathways following NEX treatment (Kwakye et al., 2023). Elevated blood glucose stimulates insulin secretion (Kwakye et al., 2023) to promote glucose uptake by cells and suppress glucose synthesis in the liver (Norton et al., 2022), which might explain the higher levels of plasma insulin associated with greater glucose in the plasma of NEX broilers.

We observed higher levels of total protein and increased circulatory globulins, including globulin, alpha 1 globulin, alpha 2 globulin, beta 1 globulin, and beta 2 globulin, in NEX broilers than in the CON treatment. Similarly, previous studies have reported that EOs such as cinnamaldehyde, eugenol, and thymol increase blood protein and globulin concentrations in broiler chickens (Amad et al., 2011; Hashemipour et al., 2014; Irawan et al., 2021). These changes in blood proteins could be due to better protein absorption and intestinal morphometric parameters following NEX treatment (Hernández et al., 2004; Hashemipour et al., 2014).

The present study demonstrated a decrease in ALT enzyme levels in the plasma of broilers fed NEX. Other studies have shown that EOs blends and brown seaweed lower circulating ALT levels in broiler chickens (Reis et al., 2018; Perali et al., 2020; Akinyemi and Adewole, 2022). The decline in plasma ALT levels in response to NEX supplementation could reflect a hepatoprotective effect because ALT is released into the bloodstream as a result of hepatocyte damages (Giannini et al., 2005; Tessari et al., 2010; Will Castro et al., 2016). Therefore, the reduction in plasma ALT concentration in the NEX treatment group might indicate better liver health compared to CON broilers.

The current study indicated that dietary NEX reduced circulatory LDL cholesterol content. This result is supported by previous studies reporting low LDL cholesterol when oregano extract and EOs such as thymol were introduced into the diet of broiler chickens (Hashemipour et al., 2014; Madkour et al., 2024c), clearly demonstrating the potential hypocholesterolemic effect of NEX supplementation in broiler chickens. This hypolipidemic effect of dietary NEX may be mediated by the stimulation of lipid catabolism during NEX treatment (Tsai et al., 2014). In support of this notion, NEX birds upregulated *PPARA* and *MTTP* genes in liver. *PPARA* stimulates lipolysis by upregulating acyl-coenzyme A oxidase and carnitine palmitoyltransferase, leading to the induction of mitochondrial β-oxidation and microsomal ω-oxidation (Reddy, 2001; Ding et al., 2003; Tu et al., 2024). *PPARA* depletions in the liver are associated with lipid accumulation in the liver (Régnier et al., 2018). The activation of *PPARA* suppresses adiposity and circulating lipids in mice fed a high-fat diet (Goto et al., 2011). Upregulation of hepatic *MTTP* can improve overall metabolic health by ensuring efficient lipid transport and utilization, which can have positive effects on energy balance and glucose metabolism (Iqbal et al., 2020). Although the current study indicated that NEX supplementation enhanced lipase activity and promoted epithelial development in the small intestine, potentially increasing circulating fatty acids, the upregulation of hepatic *PPARA* and *MTTP* likely facilitated lipid degradation and reduced LDL cholesterol in the plasma of NEX broilers. This process possibly generated more available energy in the liver, thereby supporting the greater nutrient efficiency in the NEX treatment.

This study is the first to investigate the synergistic effects of a microencapsulated blend of EOs (cinnamaldehyde, eugenol, and thymol) and seaweed meal (*Ascophyllum nodosum*) on growth performance, gut health, and metabolic functions in broiler chickens. While previous studies have explored the individual effects of EOs or seaweed supplements, the combination of these additives in a microencapsulated form has not been extensively studied. The use of microencapsulation technology ensures targeted release of the active compounds in the small intestine, maximizing their bioavailability and efficacy. This novel approach offers a promising alternative to antibiotic growth promoters in poultry production.

## Conclusions

The microencapsulated essential oil–seaweed blend (NEX) demonstrated substantial benefits for broiler chickens, including improved feed efficiency, reduced mortality, and enhanced growth performance for 35 d. NEX supplementation also led to better economic outcomes as reflected by a higher EPEF, potentially indicating greater profitability and operational efficiency. Physiological improvements, such as increased intestinal enzyme activity, better gut morphology, and enhanced mucosal integrity, were observed, supporting NEX’s role in boosting nutrient utilization and metabolic health. The upregulation of genes related to nutrient absorption and lipid metabolism implies the mechanisms behind these benefits. NEX is therefore a promising dietary supplement for optimizing broiler growth, health, and economic viability during poultry production. While this study provides valuable insights into the effects of a microencapsulated blend of EOs and seaweed meal on broiler performance, it has some limitations. The use of a single dose and the lack of individual component testing limit our ability to fully understand the contributions of each additive and their potential synergies. Future studies should include dose–response experiments, as well as treatments testing the individual components (EOs alone, seaweed meal alone) and their combinations, to provide a more comprehensive understanding of their effect. Future studies should explore the long-term effects of NEX supplementation, investigate its cost-effectiveness, and evaluate its potential in other poultry species or under different environmental conditions. Additionally, further research is needed to elucidate the mechanisms underlying the synergistic effects of microencapsulated EOs and seaweed meal on gut health and metabolic functions.

## Abbreviations

ADG: average daily gain
AFRH: the Applied Feed Research House
AGPs: antimicrobial growth promoters
ALP: alkaline phosphatase
ALT: alanine transaminase
AST: aspartate aminotransferase
BW: live body weight
Ct: cycle threshold
EOs: essential oils
EPEF: European production efficiency factor
*FASN*: fatty acid synthase gene
FCR: feed conversion ratio
FI: average daily feed intake
HDL: high-density lipoprotein cholesterol
IBW: the initial body weight
LDL: low-density lipoprotein cholesterol
*MTTP*: microsomal triglyceride transfer protein gene
*MUC2*: mucin2 gene
*PPARA*: peroxisome proliferator-activated receptor alpha gene
*PPARG*: peroxisome proliferator-activated receptor gamma gene
qRT-PCR: quantitative real-time polymerase chain reaction
*SCD*: stearoyl-CoA desaturase gene
*SLC15A1*: solute carrier family 15 member 1 gene
*SLC1A1*: solute carrier family 1 member 1 gene
*SLC2A2*: solute carrier family 5 member 1 gene
*SLC5A1*: solute carrier family 5 member 1 gene
*SLC7A1*: solute carrier family 7 member 1 gene
VLDL: very low-density lipoprotein cholesterol
