# Exression of Concern: Effects of microencapsulated essential oils and seaweed meal on growth performance, digestive enzymes, intestinal morphology, liver functions, and plasma biomarkers in broiler chickens

**DOI:** 10.1093/jas/skag112

**Published:** 2026-05-26

This is an expression of concern regarding: Ahmed A Elolimy, Mosaad M Hashim, Salah A Elsafty, Abdel Rahman Y Abdelhady, Stéphanie Ladirat, Mohamed Shourrap, Mahmoud Madkour, Effects of microencapsulated essential oils and seaweed meal on growth performance, digestive enzymes, intestinal morphology, liver functions, and plasma biomarkers in broiler chickens, *Journal of Animal Science*, Volume 103, 2025, skaf092, https://doi.org/10.1093/jas/skaf092

Following publication of this article, concerns were raised with the Editor-in-Chief regarding the integrity and reliability of aspects of the work reported in this paper. These matters are currently under investigation. The journal is investigating these concerns in line with COPE guidance. The journal is publishing this Expression of Concern to alert readers while the investigation is ongoing and advises readers to examine the details of this study with particular care.

